# Calcium nutrition nanoagent rescues tomatoes from mosaic virus disease by accelerating calcium transport and activating antiviral immunity

**DOI:** 10.3389/fpls.2022.1092774

**Published:** 2022-12-06

**Authors:** Shuo Yan, Qian Hu, Ying Wei, Qinhong Jiang, Meizhen Yin, Min Dong, Jie Shen, Xiangge Du

**Affiliations:** ^1^ Department of Plant Biosecurity and MARA Key Laboratory of Surveillance and Management for Plant Quarantine Pests, College of Plant Protection, China Agricultural University, Beijing, China; ^2^ Development Center for Science and Technology, Ministry of Agriculture and Rural Affairs, Beijing, China; ^3^ State Key Laboratory of Chemical Resource Engineering, Beijing Lab of Biomedical Materials, Beijing University of Chemical Technology, Beijing, China

**Keywords:** calcium nutrition, calcium transport, nano-fertilizer, nutrient-use efficiency, polymer

## Abstract

As an essential structural, metabolic and signaling element, calcium shows low remobilization from old to young tissues in plants, restricting the nutrient-use efficiency and control efficacy against mosaic virus disease. Nanotechnology has been applied to prevent/minimize nutrient losses and improve the accessibility of poorly-available nutrients. Herein, the current study applied a star polycation (SPc) to prepare a calcium nutrition nanoagent. The SPc could assemble with calcium glycinate through hydrogen bond and Van der Waals force, forming stable spherical particles with nanoscale size (17.72 nm). Transcriptomic results revealed that the calcium glycinate/SPc complex could activate the expression of many transport-related genes and disease resistance genes in tomatoes, suggesting the enhanced transport and antiviral immunity of SPc-loaded calcium glycinate. Reasonably, the calcium transport was accelerated by 3.17 times into tomato leaves with the help of SPc, and the protective effect of calcium glycinate was remarkably improved to 77.40% and 67.31% toward tomato mosaic virus with the help of SPc after the third and fifth applications. Furthermore, SPc-loaded calcium glycinate could be applied to increase the leaf photosynthetic rate and control the unusual fast growth of tomatoes. The current study is the first success to apply nano-delivery system for enhanced calcium transport and antiviral immunity, which is beneficial for increasing nutrient-use efficiency and shows good prospects for field application.

## Introduction

Calcium is an essential structural, metabolic and signaling element, which is required either for nutritional or signaling purpose (Demidchik et al., 2018; Thor, 2019). It thereby shows a dual function, both as a structural component of cell walls and membranes and as a second messenger, which is involved in plant growth and responses to abiotic as well as biotic stress (Knight et al., 1997; Cheong et al., 2002; Monshausen et al., 2011; Xu et al., 2015; Zhang et al., 2017). Calcium is mainly present in soil solution, and low soil availability of calcium is usually not the reason for calcium deficiency (White and Broadley, 2003). Deficiency symptoms are usually observed in developing tissues such as young leaves and fruits, due to the low remobilization of calcium from old to young tissues. Symplastic pathway is mainly used for short-distance calcium delivery, and the long-distance translocation is dependent on apoplastic pathway *via* xylem (Jovanović et al., 2021). Transpiring organs tend to accumulate high calcium levels, and calcium is usually deposited inside the vacuoles or sequestrated into leaf-trichomes to impose calcium phloem immobility, which leads to low calcium levels in young leaves and fruits (White, 2001; Gilliham et al., 2011; Kumar et al., 2015). This property leads to low nutrient-use efficiency of calcium and further restricts its antiviral immunity toward devastating mosaic virus disease. Thus, efficient delivery of calcium into plant young tissues is crucial for calcium bioavailability, especially for alleviating mosaic virus disease that is regarded as “tomato cancer”.

Achieving sustainable agricultural productivity and global food security requires innovative technologies during the Green Revolution. One of concerns is the inefficient overuse of agrochemicals, and nanotechnology has the potential to boost agricultural industry through their nanospecific properties (Wang et al., 2022a). Nanocarriers have been applied to prevent/minimize nutrient losses, allow the controlled-release of nutrients, and improve the accessibility of poorly-available nutrients (Chhipa, 2016; Zuverza-Mena et al., 2017; Jakhar et al., 2022). Nano-sized nutrients can increase the surface mass ratio of fertilizers, which allows a remarkable enhancement of root absorption. With the help of nanocarriers, slow, targeted and efficient nutrient release becomes possible, which can reduce application dosage/cost to minimize nutrient losses and increase nutrient-use efficiency (Fellet et al., 2021). During the last decade, great attention has been paid to calcium nanoparticles as potential nano-fertilizers with superior nutrient-use efficiency compared to their conventional counterparts (Carmona et al., 2022). Previous study reports a nano calcium phosphate that can significantly increase the shoot and root dry weights, nutrient content, yield components, and nutrient concentration and protein percentage in the pods of snap bean plants (El-Ghany et al., 2021).

Our studying group has designed and constructed a star polycation (SPc)-based nano-delivery system that can be applied to deliver various exogenous substances such as double-stranded RNA (dsRNA) and synthetic/botanical pesticides (Li et al., 2019a; Yan et al., 2021a; Yan et al., 2021b; Ma et al., 2022a; Zhang et al., 2022a). The SPc-based nano-delivery system can reduce the particle size of synthetic/botanical pesticides and increase their water solubility and dispersity (Yan et al., 2021b; Yan et al., 2022; Wang et al., 2022b). At the cellular level, the SPc can activate the clathrin-mediated endocytosis for enhanced cellular uptake and promote the endosomal escape for intracellular spreading of exogenous substances (Wang et al., 2021; Ma et al., 2022b; Yang et al., 2022; Zhang et al., 2022b). *In vivo*, the SPc can increase plant uptake to improve the bioactivity of various exogenous substances (Wang et al., 2022b; Jiang et al., 2022a; Jiang et al., 2022b). A recent publication has demonstrated that the SPc can co-deliver dsRNA and matrine to overcome the short life disadvantage of dsRNA and slow-acting property of matrine simultaneously (Li et al., 2022). Applying SPc-based nano-delivery system for efficient plant uptake of nutrients is a new topic.

Calcium glycinate (C_4_H_8_CaN_2_O_4_) is formed by glycine and calcium compounds through chemical reactions, which is a new-type and ideal nutrient supplement (Yin et al., 2017). To this context, the current study aimed to construct a SPc-based nano-delivery system for calcium glycinate to control tomato mosaic virus (ToMV) disease by accelerating calcium transport and activating antiviral immunity. We tested the drug loading content (DLC) using freeze drying method, measured the complex particle size using dynamic light scattering (DLS), observed the complex morphology using transmission electron microscope (TEM), and determined the interaction force between calcium glycinate and SPc using isothermal titration calorimetry (ITC) to illustrate the self-assembly mechanism. Then, we determined the differentially expressed genes (DEGs) between calcium glycinate/SPc complex and calcium glycinate alone to explore the potential function of SPc. Finally, we tested the calcium transport in plant with the aid of SPc using flame atomic absorption spectrometry (FAAS) system, and determined the bioactivity of SPc-loaded calcium glycinate, including the control efficacy against ToMV, and effects on leaf photosynthetic rate and internodes length of seedlings.

## Materials and methods

### Chemicals and SPc synthesis

Pure calcium glycinate (≥98%) was purchased from Jiangsubaiye Biotechnology Co., Ltd (Nanjing, China). N,N,N′,N′,N″-Pentamethyl diethylenetriamine (PMDETA, 98%) and CuBr (99.999%) purchased from Sigma-Aldrich (Saint Louis, MO, USA), 2-bromo-2-methylpropionyl bromide and triethylamine purchased from Heowns BioChem Technologies (Tianjin, China), and 2-(Dimethyl amino) ethyl methacrylate (DMAEMA, 99%) purchased from Energy Chemical (Shanghai, China) were used for SPc synthesis. Other chemical reagents such as ethanol, methanol, etc. were purchased from Beijing Chemical Works (Beijing, China).

SPc was synthesized through two reaction steps following a previously described method (Li et al., 2019a). Briefly, the star initiator Pt-Br was constructed by adding 2-bromo-2-methylpropionyl bromide (253 mg, 1.11 mmol) dropwise into pentaerythritol solution (25 mg, 0.18 mmol) in dry tetrahydrofuran (20 mL) and triethylamine (111.3 mg, 1.11 mmol) at 0°C, and stirring for 24 h at room temperature. The reaction was quenched by adding methanol, and obtained Pt-Br was polymerized with DMAEMA (2.2g, 7.7 mmol) in an oil bath at 60°C for 7 h with the help of tetrahydrofuran (8 mL), PMDETA (110 mg, 0.44 mmol) and CuBr (46 mg, 0.22 mmol). The reaction was quenched by cooling and air exposure, and dialysis was then carried out to purify the crude product of SPc. The SPc was dissolved in double distilled water (ddH_2_O) to prepare the 60 mg/mL stock.

### Loading capacity measurement

DLC was tested according to previous described methods (Yan et al., 2022; Wang et al., 2022b). Briefly, excess calcium glycinate (603.5 mg) was mixed with SPc (229.26 mg) in ddH_2_O (23 mL). The mixture was dialyzed using the regenerated cellulose with a molecular weight cut off of 1 000 Da (Shanghai Yuanye Bio-Technology Co., Shanghai, China) for 12 h, freeze-dried and weighed. DLC was calculated as DLC (%) = weight of calcium glycinate loaded in complex/weight of calcium glycinate – loaded complex × 100%.

### Isothermal titration calorimetry (ITC) assay

The SPc and calcium glycinate were dissolved in ddH_2_O, respectively. The 300 μL calcium glycinate (monomer content: 200 μmol/L) was titrated with 40 μL SPc aqueous solution (250 μmol/L) in MicroCal iTC200 (GE Healthcare Life Sciences, MA, USA). During each injection, the heating temperature of interaction was calculated by integrating each titration peak *via* Origin7 software (OriginLab Co., Ltd., MA, USA). The test temperature was set at 25°C, and the ΔG value was calculated using the formula of ΔG = ΔH – TΔS.

### Particle size measurement and morphology observation

Calcium glycinate was dissolved in ddH_2_O, and mixed with SPc at the mass ratio of 1:8.8 to prepare calcium glycinate/SPc complex (1 mg/mL) according to the DLC. The calcium glycinate was too big for particle size measurement, thus only calcium glycinate/SPc complex was measured using Particle Sizer and Zeta Potential Analyzer (Brookhaven NanoBrook Omni, New York City, NY, USA) at 25°C. The particle size of calcium glycinate/SPc complex was also measured after 14 d storage to test the complex stability. Each assay was repeated 3 times. Furthermore, morphological characteristics of calcium glycinate and calcium glycinate/SPc complex were observed using TEM (JEOL-1200, Japan). A few microliters of each sample were dropped on microgrid, treated with 2% phosphotungstic acid, and air-dried before the observation.

### RNA-seq analysis and quantitative real time PCR (qRT-PCR) for gene expression changes

Tomato (*Solanum lycopersicum*) seedlings were cultured in growth chamber at 26°C under a 12-h light: 12-h dark photoperiod. The roots of 15 cm height seedlings were immersed in ddH_2_O for 48 h, and then transferred to the solutions of calcium glycinate (0.4 mg/mL) and calcium glycinate/SPc complex (0.4 mg/mL at the mass ratio of 1:1) respectively. The whole seedlings were homogenized in liquid nitrogen at 3 d post immersion, and total RNAs were isolated using RNA simple Total RNA Kit (Tiangen, Beijing, China). Each treatment included 3 biological replicates.

RNA sequencing libraries were constructed and then sequenced on Illumina Hiseq platform (Biomics, Beijing, China). Raw data were purified by trimming the adapters and removing low quality reads (Q ≤ 10), and the clean reads were assembled into contigs using Trinity software (Grabherr et al., 2011). Assembled transcripts were aligned to the reference genome (http://plants.ensembl.org/Solanum_lycopersicum/Info/Index) using TopHat2 (Kim et al., 2013). The expression level of each transcript was presented by FPKM values. The DEGs were analyzed using DESeq2 R package, and the genes with fold change ≥2 and with false discovery rate <0.01 were considered to be differentially expressed (Love et al., 2014).

QRT-PCR was further carried out to verify the DEGs using the primers in **Table S1**. Total RNAs were extracted from calcium glycinate or calcium glycinate/SPc complex-exposed seedlings, and the cDNAs were synthesized using Hifair First Strand cDNA Synthesis Kit (Yeasen Biotech, Shanghai, China). The qRT-PCR was conducted on ABI QuantStudio 6 Flex System (Thermo Fisher, MA, USA) using Perfect Start Green qPCR Super Mix (TransGen Biotech, Beijing, China). Reactions were carried out in triplicate following the amplification protocols: one cycle at 95°C for 10 min, 40 cycles of 95°C for 15 s, 57°C for 30 s, and 72°C for 35 s, and a melting curve ramp to confirm that each reaction did not produce nonspecific amplification. The *actin* gene was used as the internal control for qRT-PCR, and the gene expression level was calculated using 2^-ΔΔCt^ method (Livak and Schmittgen, 2001).

### Calcium transport assay

Fifty cm height tomato seedlings were cultured in ddH_2_O for 48 h, and then transferred in the solutions of calcium glycinate and calcium glycinate/SPc complex similarly as above. The ddH_2_O and SPc were also employed as controls. Each treatment included 3 independent samples. At 24 h after the treatment, all leaves were collected, dried and homogenized. The 0.2 g leaf tissues were added with 8 mL nitric acid, incubated for 3 h, and treated using microwave digestion system (MARS-6, CEM, Matthews, USA) that comprised a power system with selectable output of 0-1200 W. The heating program was (i) 1200 W for 60 min and (ii) 0 W for 25 min (cooling). The above samples were diluted with ddH_2_O to the volume of 50 mL, and 10 mL sample was used to determine calcium content using the flame atomic absorption spectrometry (FAAS) system (TRACE AI1200, AURORA Instruments, Vancouver, Canada) equipped with deuterium lamp for background correction system. The lamp operated at 10 mA (wavelength 422.7 nm) for calcium content measurement. The calcium transport was calculated using the formula of calcium transport = calcium content in each treatment – calcium content in ddH_2_O treatment. The standard calibration curve was constructed using CaCO_3_. The 2.597 g CaCO_3_ was dissolved in 100 mL H_2_O, and added with 10 mL nitric acid to prepare the stock. The stock was further diluted with 5% nitric acid to prepare a series of CaCO_3_ dilutions (Ca concentration: 10, 20, 40, 60, 80 and 100 mg/L), and the calcium content was examined similarly as above.

### Bioactivity assay of SPc-loaded calcium glycinate

The bioactivity of SPc-loaded calcium glycinate was evaluated in a tomato greenhouse (Haotianyuan farm, Beijing, China) with a day/night temperature of 25/18°C. According to the field layout (**Figure S1**), the area of each plot was approximately 12 m^2^, and each plot contained 30-40 seedlings. Each treatment included 3 independent plots. The formulations of calcium glycinate/SPc complex (0.8 and 0.4 mg/mL at the mass ratio of 1:1, 80% and 40% recommended field concentration), calcium glycinate (0.8 and 0.4 mg/mL), SPc (0.8 and 0.4 mg/mL) and H_2_O were sprayed against tomato seedlings every 10 days for 5 times with the application amount of 140 mL/m^2^.

The disease indexes (DIs) of seedlings treated with calcium glycinate/SPc complex (40% recommended concentration) and calcium glycinate were evaluated toward ToMV at 10 d after the third, fourth and fifth applications, respectively. All seedlings from each plot were observed to record disease grades that were classified as follows. Grade 0: no symptoms; Grade 1: mild malformation and mosaic of spear leaf; Grade 2: lesion area of all leaves was less than 1/3; Grade 3: leaf malformation and mosaic in all leaves; Grade 4: all leaves showing severe mosaic, dwarfing and malformation. DI and protective effect (PE) were calculated using the following formulas.


$$\text{DI}=\frac{\sum^{}\left(\text{number of diseased plants}\times \text{grade}\right)}{\left(\text{number of investigated plants}\times 4\right)}\times 100$$

$$\text{PE}=\left(1\;-\;\frac{\text{DI in treatment plot}}{\text{DI in control plot}}\right)\times 100\%$$

Leaf photosynthetic rate and internodes length of seedlings were also measured at 10 d post the first and second application. According to the previous method (Zhang et al., 2021), leaf photosynthetic measurements were performed on fully expanded leaves (the second leaf from the top) at 9:00-10:00 a.m. using portable photosynthesis system (Shijiazhuang Shiya Technology, Co., Ltd, Shijiazhuang, China). Each leaf was allowed sufficient time for equilibration in chamber, and 5 leaves were tested in each plot. Internodes length was measured from the third to fourth leaf, and each plot contained 5 independent seedlings.

### Data analysis

Statistical analysis was carried out using SPSS 26.0 software (SPSS Inc., New York, USA). Descriptive statistics are shown as the mean value and standard errors of the mean. Data was analyzed using the one-way ANOVA with Tukey HSD test or independent *t* test at the *P* = 0.05 level of significance.

## Results

### Loading capacity and self-assembly of calcium glycinate/SPc complex

**Figure 1:**
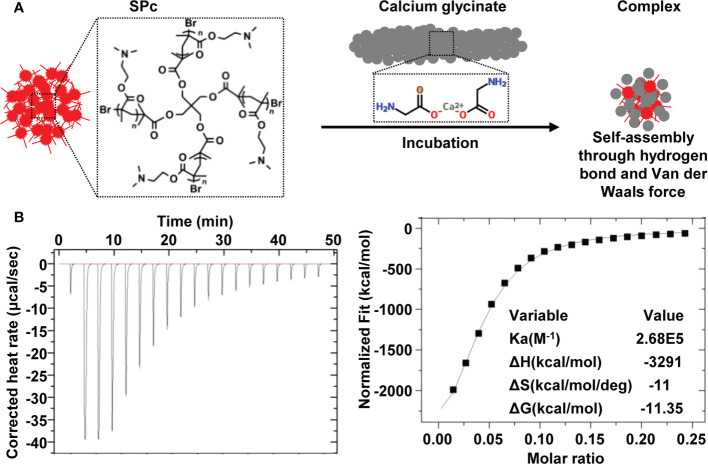
Schematic illustration of calcium glycinate/SPc complex **(A)** and ITC titration of SPc (250 μmol/L) into calcium glycinate solution (200 μmol/L) **(B)**.

The SPc could spontaneously combine with calcium glycinate into calcium glycinate/SPc complex in aqueous solution. The added calcium glycinate was excess for assembling with SPc, and the dialysis could separate free calcium glycinate from the mixture. The weight of freeze-dried sample was 255.3 mg (calcium glycinate/SPc complex), revealing that the weight of loaded calcium glycinate was 26.04 mg. The DLC was calculated to be 10.2%. Furthermore, the interaction forces between SPc and calcium glycinate was analyzed by ITC (**Figure 1**). The high affinity constant (K_a_) of 2.68×10^5^ suggested that there was a strong interaction between SPc and calcium glycinate, and the negative ΔG value of -11.35 kcal/mol revealed that this interaction was automatic. The negative values of ΔH and ΔS suggested that the self-assembly of calcium glycinate/SPc complex was through hydrogen bond and Van der Waals force.

### Characterization of calcium glycinate/SPc complex

**Figure 2:**
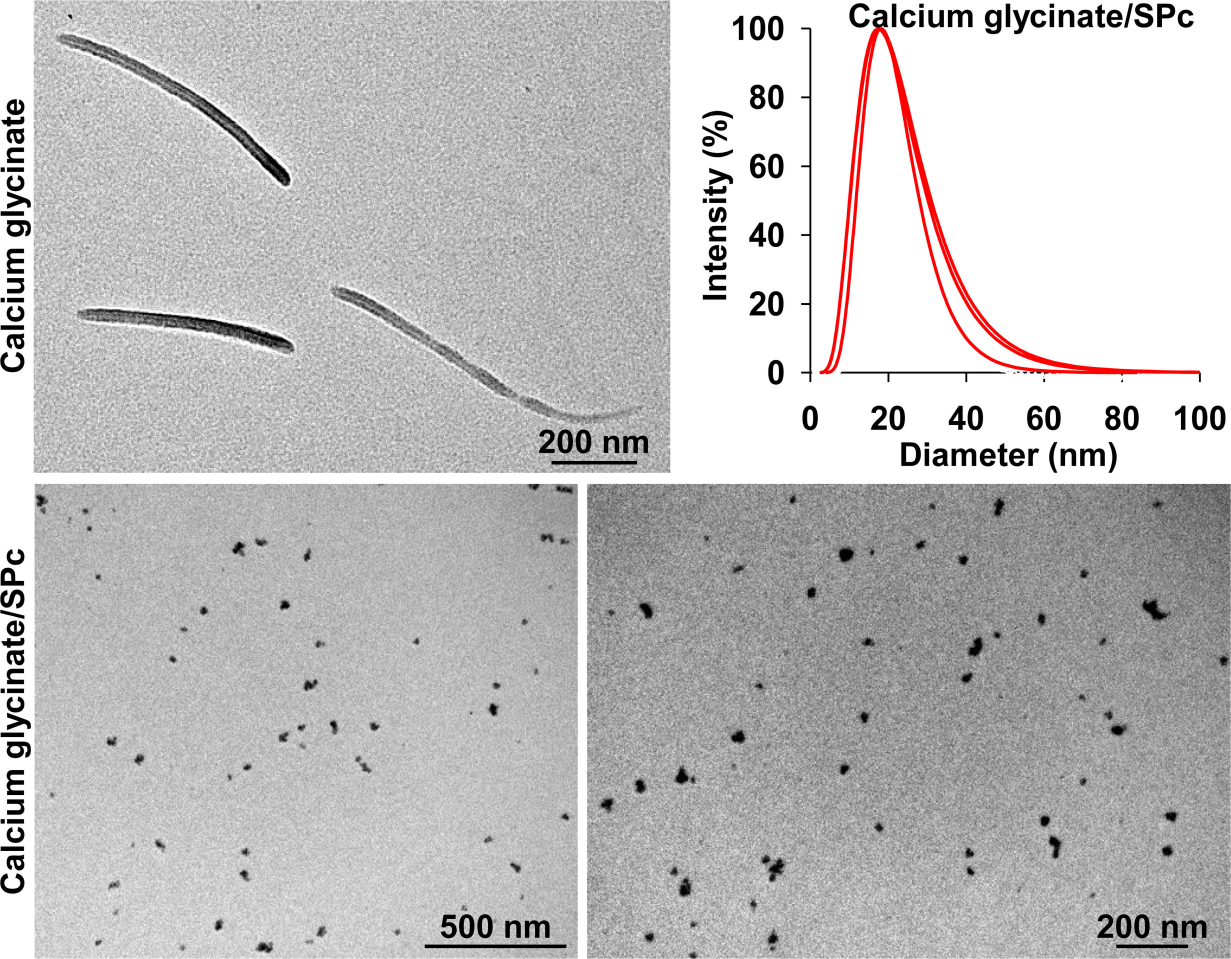
Transmission electron microscope image and particle size distribution of calcium glycinate/SPc complex at the mass ratio of 1:8.8.

As shown in **Figure 2** and **Table 1**, the self-assembly of calcium glycinate/SPc complex disturbed the self-aggregated structure of calcium glycinate, decreasing its particle size down to 17.7 nm. Meanwhile, small polydispersity value revealed good dispersity and stability of calcium glycinate/SPc complex in aqueous solution. Representative TEM images showed that the most of self-aggregated calcium glycinate was composed of stable rod-shaped particles, whereas the morphological characteristics of SPc-loaded calcium glycinate was remarkably changed, which was the spherical particle with much smaller size. Furthermore, the particle size of calcium glycinate/SPc complex was relatively stable after 14 d storage at room temperature.

**Table 1:** Particle size and polydispersity of SPc-loaded calcium glycinate before and after the storage.

Condition	Sample number	Polydispersity	Average polydispersity	Size (nm)	Average size (nm)
Before storage	1	0.147		18.10	
	2	0.244	0.215 ± 0.034	17.39	17.72 ± 0.21
	3	0.255		17.68	
After 14 d storage	1	0.241		17.70	
	2	0.245		0.239 ± 0.004		17.90	17.83 ± 0.07
	3	0.232	17.89				
			*t* = 0.695, df = 2.050, *P* = 0.557		*t* = 0.494, df = 4, *P* = 0.648

The independent t test was used to analyze the data at P = 0.05 level of significance.

### Gene expression changes induced by calcium glycinate/SPc complex

**Figure 3:**
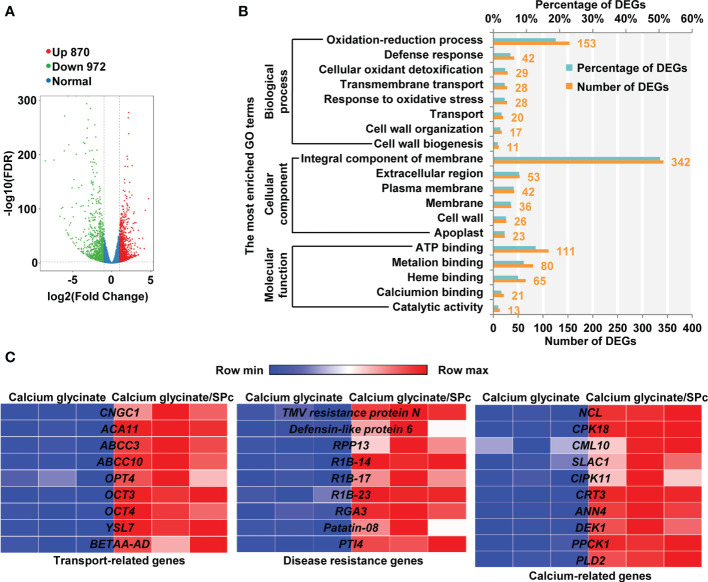
RNA-seq analysis of tomatoes treated with calcium glycinate and calcium glycinate/SPc complex. **(A)** Analysis of differentially expressed genes (DEGs) in tomatoes with a volcano plot. Up-regulated and down-regulated DEGs are represented by red and green dots, respectively. **(B)** GO enrichment analysis of DEGs in biological process, cellular component and molecular function. **(C)** Heatmaps of differentially expressed genes. Highly and lowly expressed genes are labeled as red and blue, respectively. Gene names are listed in the middle.

Transcriptomic analysis was performed to illustrate the plant responses upon calcium glycinate/SPc complex exposure. The RNA samples had high sequencing quality (**Table S2**) and good parallelism (**Figure S2**). Compared to calcium glycinate/SPc complex, a total of 1842 DEGs were identified in tomatoes upon calcium glycinate exposure, among which 870 genes were up-regulated and 972 genes were down-regulated (**Figure 3A**). The DEGs could be divided into three categories including biological process, cellular component and molecular function, and GO enrichment analysis revealed that several signaling pathways related with defense response, transport, membrane, cell wall and calcium were strongly influenced (**Figure 3B**).

Typical DEGs with calcium glycinate/SPc complex exposure are shown in **Figure 3C**. Compared to calcium glycinate alone, calcium glycinate/SPc complex up-regulated many transport-related genes of tomatoes, such as *cyclic nucleotide-gated ion channel 1* (*CNGC1*), *putative calcium-transporting ATPase 11, plasma membrane-type* (*ACA11*), *ABC transporter C family member 3* (*ABCC3*), *ABCC4*, *oligopeptide transporter 4* (*OPT4*), etc. The up-regulation of these genes suggested the enhanced delivery of calcium glycinate with the help of SPc. Meanwhile, the SPc-loaded calcium glycinate also activated the expression of multiple disease resistance genes, such as *TMV resistance protein N*, *defensing-like protein 6*, *disease resistance protein RPP13* (*RPP13*), *putative late blight resistance protein homology R1B-14* (*R1B-14*), etc. The up-regulation of these genes suggested that the SPc-loaded calcium glycinate could further activate plant systemic immunity to resist adverse stresses. Furthermore, the expression of some other calcium-related genes was significantly altered by SPc-based nano-delivery system. More specifically, *sodium/calcium exchanger NCL* (*NCL*), *calcium-dependent protein kinase 18* (*CPK18*), *Probable calcium-binding protein CML10* (*CML10*), *Guard cell S-type anion channel SLAC1* (*SLAC1*), etc. were remarkably up-regulated in tomato seedlings treated with SPc-loaded calcium glycinate. The quantitative real time PCR (qRT-PCR) analysis revealed that the expression levels of above genes were in accordance with transcriptome results (**Figure S3**). More specifically, expressions of *CNGC1*, *ACA11* and *TMV resistance protein N* genes were up-regulated by 5.13, 2.70 and 2.88-fold, respectively.

### Enhanced transport of SPc-loaded calcium glycinate

**Figure 4:**
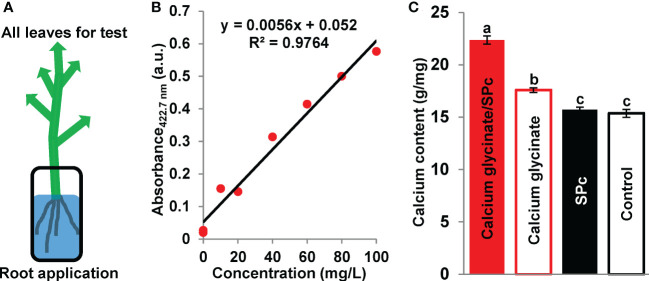
Enhanced transport of SPc-loaded calcium glycinate. **(A)** Schematic diagram. Tomato seedlings were cultured in the solutions of calcium glycinate and calcium glycinate/SPc complex for 24 h. All leaves were collected, dried and homogenized for calcium content measurement. **(B)** Standard calibration curve of calcium. **(C)** Calcium content in leaves. Each treatment included 3 independent samples. Different letters indicate significant differences according to Tukey HSD test (*P* < 0.05).

Whether the nano-sized calcium glycinate exhibits stronger transport property is an interesting topic. The current study demonstrated that the SPc could promote the transport of calcium glycinate to increase the calcium content in tomato leaves (**Figure 4**). More specifically, the calcium content was increased from 17.58 to 22.38 g/mg with the help of SPc, and the calcium transport was accelerated by 3.17 times. Plant uptake and bioaccumulation are directly related with the bioactivity of calcium glycinate.

### Enhanced bioactivity of SPc-loaded calcium glycinate

**Figure 5:**
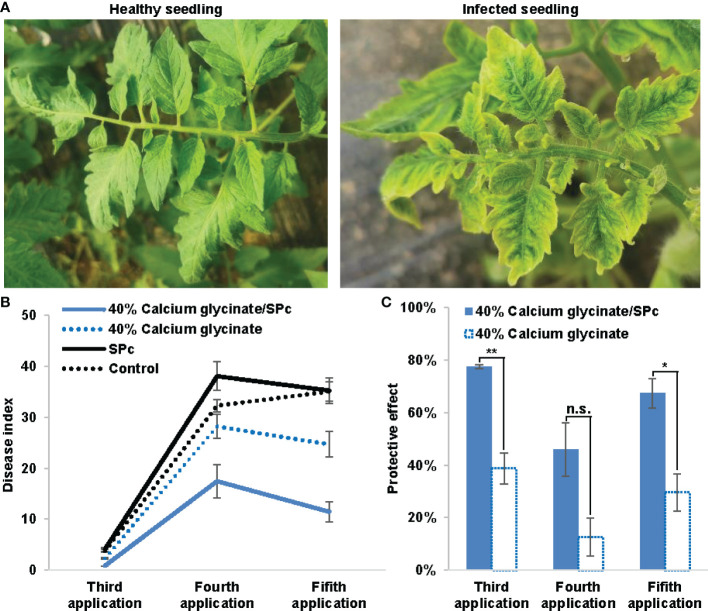
Enhanced bioactivity of SPc-loaded calcium glycinate toward tomato mosaic virus. **(A)** Photos of healthy and infected seedlings. **(B)** Disease indexes of seedlings were evaluated at 10 d post the third, fourth and fifth applications, respectively. All seedlings from each plot were observed to record the disease grades. **(C)** Protective effect was assessed by disease index. The “*” and “**” indicate significant differences (Independent *t* test, *P* < 0.05 and *P* < 0.01), and “n.s.” indicates no significant difference.

The DIs of tomato seedlings sprayed with calcium glycinate/SPc complex and calcium glycinate were firstly evaluated and compared toward ToMV (**Figure 5**). Due to the SPc application, the control effect of calcium glycinate was significantly improved with DI reduction from 28.21 to 17.46 and 24.69 to 11.47 after the fourth and fifth applications, respectively. Meanwhile, the protective effect of calcium glycinate was significantly increased to 77.40% and 67.31% with the help of SPc after the third and fifth applications, revealing the good application prospect of calcium glycinate/SPc complex in field.

**Figure 6:**
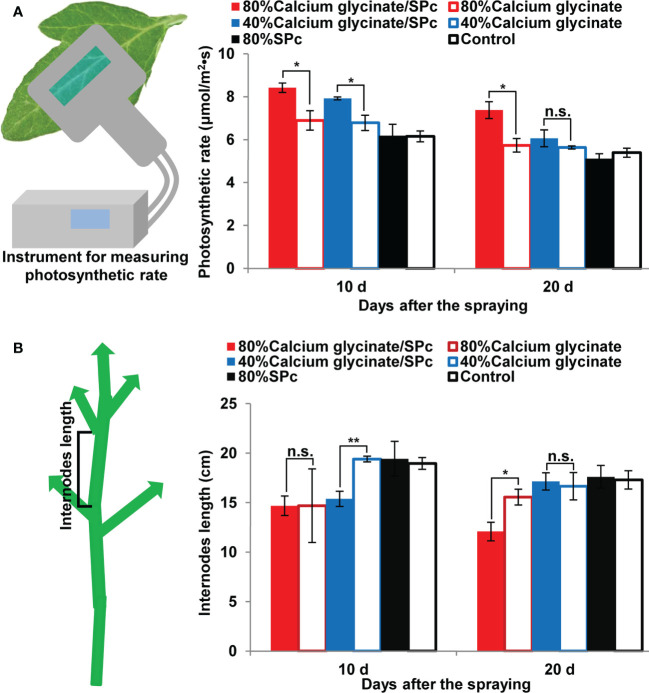
Effects of SPc-loaded calcium glycinate on the photosynthetic rate **(A)** and internodes length **(B)** of tomato leaves/seedlings. The seedlings were sprayed with various formulations at 80% and 40% recommended field concentration. The photosynthetic rate and internodes length were measured at 9:00-10:00 a.m. at 10 d post the first and second applications. Five leaves/seedlings were tested in each plot. The “*” and “**” indicate significant differences (Independent *t* test, *P* < 0.05 and *P* < 0.01), and “n.s.” indicates no significant difference.

Leaf photosynthetic rate was also measured and compared to illustrate the enhanced bioactivity of SPc-loaded calcium glycinate. As shown in **Figure 6A**, the photosynthetic rate of tomato leaves was increased with the help of SPc, ranging from 6.90 to 8.42 μmol/m^2^•s (80% concentration) and from 6.78 to 7.92 μmol/m^2^•s (40% concentration) after the first application, revealing the stronger photosynthesis of tomato leaves. Furthermore, the internodes length of tomato seedlings tended to decrease by applying calcium glycinate/SPc complex compared to calcium glycinate alone, suggesting that the SPc-loaded calcium glycinate could be applied to control the unusual fast growth of tomato plants (**Figure 6B**).

## Discussion

The SPc could spontaneously combine with calcium glycinate into calcium glycinate/SPc complex in aqueous solution. However, its DLC was lower than those toward pesticides such as imidaclothiz (16.31%), dinotefuran (17.41%) and osthole (17.09%) (Yan et al., 2021b; Jiang et al., 2022a; Jiang et al., 2022b). According to the previous interpretation of ITC data (Ross and Subramanian, 1981), the self-assembly of calcium glycinate/SPc complex was through hydrogen bond and Van der Waals force, and this interaction was automatic. The complexation of SPc with exogenous substance can be achieved *via* different interaction forces, such as electrostatic interaction with thiamethoxam (Yan et al., 2021a) and lufenuron (Zhang et al., 2022b), hydrogen bond and Van der Waals force with chitosan (Wang et al., 2021) and dinotefuran (Jiang et al., 2022a), and hydrophobic association with thiocyclam (Wang et al., 2022b). Thus, the application area of SPc is broad for delivering various types of exogenous substances.

The self-assembly of calcium glycinate/SPc complex disturbed the self-aggregated structure of calcium glycinate, decreasing its particle size down to nanoscale. The SPc has been widely applied as a universal adjuvant for various pesticides, which can decrease the particle sizes of thiamethoxam, dinotefuran and osthole in aqueous solution (Yan et al., 2021a; Yan et al., 2021b; Jiang et al., 2022a). The smaller particle size of SPc-loaded calcium glycinate may be beneficial for not only improving plant uptake and systematic transmission, but also increasing nutrient-use efficiency to minimize nutrient losses.

Calcium is involved in mediating plant responses to various adverse environmental conditions, and some transcriptome studies have confirmed the great function of calcium in multiple responses to pathogens, wounding, heat stresses, salt stresses, etc. (Cheong et al., 2002; Navarro et al., 2004; Xu et al., 2015; Wang et al., 2019a). Compared to calcium glycinate alone, calcium glycinate/SPc complex up-regulated many transport-related genes of tomatoes. For instance, CNGC1 protein is responsible for cAMP-induced calcium entry in cells, thus is involved in calcium signal transduction (Sunkar et al., 2000). CAC11 is a magnesium-dependent enzyme that can catalyze the hydrolysis of ATP coupled with the translocation of calcium from the cytosol out of the cell or into organelles (Boursiac et al., 2010). The up-regulation of these two important genes suggested the enhanced delivery of calcium glycinate with the aid of SPc. Our previous studies have demonstrated that the endocytosis-related genes of plants/insects can be activated by SPc application to increase the cellular uptake (Wang et al., 2021; Ma et al., 2022b; Yang et al., 2022). The SPc can also promote the translocation of insect growth regulator/dsRNA across insect cuticle (Yan et al., 2020; Ma et al., 2022a; Zhang et al., 2022a; Zhang et al., 2022b).

The SPc-loaded calcium glycinate also activated the expression of multiple disease resistance genes. TMV resistance protein N can trigger the defense system to restrict the mosaic virus, and it’s up-regulation leads to stronger antiviral immunity (Whitham et al., 1994; Dinesh-Kumar and Baker, 2000; Marathe et al., 2002). Defensing-like protein is a major component of plant immune system, which plays an important role in host defenses against biotic and abiotic stresses (Spelbrink et al., 2004; Wang et al., 2019b). RPP13 guards the plants against pathogens containing an appropriate avirulence protein (Bittner-Eddy et al., 2000). Furthermore, the expression of some other calcium-related genes was also altered. NCL protein has the ability to bind calcium *in vitro*, which is involved in the maintenance of calcium homeostasis and response to auxin and salt stress (Wang et al., 2012; Li et al., 2016). As key messengers in signal transduction, CPKs are involved in responses to cold stress, salt stress, wounding and drought (Liu et al., 2018; Shu et al., 2020). The up-regulation of these important genes suggested that the SPc-loaded calcium glycinate could further activate plant systemic immunity to resist adverse stresses.

Vacuole deposition of calcium imposes the low mobility in phloem, which causes deleterious nutritional disorders (Gilliham et al., 2011; Kumar et al., 2015; Jovanović et al., 2021). The first step for the plant uptake of calcium is facilitated by cation exchange in soil, then calcium is transported apoplastically to endodermis, and finally calcium reaches the xylem sap and is translocated *via* the transpiration stream to aerial plant parts (Bauer et al., 2011; Ghosh et al., 2022). The current study demonstrated that the SPc could promote the transport of calcium glycinate to increase the calcium content in tomato leaves. The enhanced plant uptake of SPc-loaded agrochemicals has been previously observed in tobacco, oilseed rape and strawberry (Yan et al., 2021b; Jiang et al., 2022a; Jiang et al., 2022b). For instance, the SPc can increase the plant uptake of a synthetic pesticide dinotefuran for 1.45-1.53 times due to the smaller particle size and reduced contact angle (Jiang et al., 2022a). Meanwhile, a plant elicitor chitosan can be efficiently delivered into potato plants to amplify the plant defense responses with the help of SPc (Wang et al., 2021). Plant uptake and bioaccumulation are directly related with the bioactivity of calcium glycinate.

Calcium is an essential element in diverse biological processes, such as disease resistance and plant development (Springer et al., 2007; Wang et al., 2015; Tian et al., 2020; Guo et al., 2021). Extensive studies have demonstrated that the calcium changes can mediate plant defense against pathogen infections, and many exogenous agents are able to increase TMV tolerance through calcium signaling pathway (Lu et al., 2010; Li et al., 2019b). Meanwhile, our transcriptome and qRT-PCR results confirmed the up-regulation of many disease resistance genes especially *TMV resistance protein N*. Thus, the DIs of tomato seedlings sprayed with calcium glycinate/SPc complex and calcium glycinate were evaluated and compared toward ToMV. Due to the SPc application, the control effect of calcium glycinate was improved, indicating the good application prospect of calcium glycinate/SPc complex in field.

Previous studies have revealed that the exogenous calcium application can improve plant photosynthesis. For instance, the tobacco photosynthesis can be improved by CaCl_2_ application under high temperature stress (Tian et al., 2011). Seed priming with CaCl_2_ before germination can improve the photosynthesis of faba bean plants under cadmium stress (Nouairi et al., 2019). In the current study, the SPc application could further increase the photosynthesis of tomato plants induced by calcium glycinate. The potential mechanism of SPc-mediated bioactivity enhancement could be explained as follows. The SPc-based nano-delivery system decreased the particle size of calcium glycinate in aqueous solution, thus increasing the delivery/plant uptake of calcium glycinate for enhanced bioactivity. The SPc can act as a universal nanocarrier to deliver various agrochemicals for high utilization efficiency.

## Conclusion

In summary, the current study constructed a SPc-based nano-delivery system to load calcium glycinate through hydrogen bond and Van der Waals force. The self-assembly of calcium glycinate/SPc complex formed stable spherical particles with nanoscale size (17.7 nm). Compared to calcium glycinate, calcium glycinate/SPc complex activated the expression of many transport-related genes and disease resistance genes in tomatoes, suggesting the enhanced transport and bioactivity of SPc-loaded calcium glycinate. As expected, the calcium content in tomato leaves was increased from 17.58 to 22.38 g/mg with the help of SPc, and the control effect of calcium glycinate was significantly improved toward ToMV with DI reduction from 24.69 to 11.47 after the fifth application. Furthermore, SPc-loaded calcium glycinate could be applied to increase the leaf photosynthetic rate and control the unusual fast growth of tomatoes. The current calcium nutrition nanoagent can be sprayed directly to plant leaves for increasing nutrient-use efficiency and antiviral immunity in actual production.

## Data availability statement

The original contributions presented in the study are publicly available. This data can be found here: NCBI, PRJNA907413.

## Author contributions

SY and QH contributed equally to this study. SY, JS, and XD designed the research. SY, QH, YW, QJ, and MD performed the research. All authors analyzed the date. SY wrote the paper.
