# Supplementary material for: Calcium nutrition nanoagent rescues tomatoes from mosaic virus disease by accelerating calcium transport and activating antiviral immunity
Source: Front Plant Sci. 2022 Dec 6;13:1092774. doi: 10.3389/fpls.2022.1092774 (PMC9764000; doi:10.3389/fpls.2022.1092774)

**Supplementary Information**

**Calcium nutrition nanoagent rescues tomatoes from mosaic virus disease by accelerating calcium transport and activating antiviral immunity**

Shuo Yan^1†^, Qian Hu^2†^, Ying Wei^1^, Qinhong Jiang^1^, Meizhen Yin^3^, Min Dong^1^, Jie Shen^1*^ and Xiangge Du^1*^

^1^Department of Plant Biosecurity and MARA Key Laboratory of Surveillance and Management for Plant Quarantine Pests, College of Plant Protection, China Agricultural University, Beijing 100193, P. R. China, ^2^Development Center for Science and Technology, Ministry of Agriculture and Rural Affairs, Beijing 100176, P. R. China, ^3^State Key Laboratory of Chemical Resource Engineering, Beijing Lab of Biomedical Materials, Beijing University of Chemical Technology, Beijing 100029, P. R. China.

^†^Shuo Yan and Qian Hu have contributed equally to this work

^*^Correspondence: duxge@cau.edu.cn (X. Du); shenjie@cau.edu.cn (J. Shen)

**Table S1.** Primers for quantitative real-time PCR.

| **Gene** | **Primer** | **Sequence** |
| --- | --- | --- |
| *CNGC1* | Forward primer | CCAAGGCACACGAGGAGTTG |
|  | Reverse primer | GCAGAACCTGAAATAGCCAAGT |
| *ACA11* | Forward primer | CAGGTGTTTATTGCTTTGATTGAGT |
|  | Reverse primer | TCTGTGAGTGTTGATTCGTTGA |
| *TMV resistance protein N* | Forward primer | AGCGTTATTCATCTTGTTTCAGTAA |
|  | Reverse primer | ACTCATCTTCAACCCATTTCATTT |
| *Defensin-like protein 6* | Forward primer | TTAGGTGTGGTGATGGGACG |
|  | Reverse primer | ACAATCTTCTGCTTTCTTACAGC |
| *RPP13* | Forward primer | AGGGAGAACCATCCTAAGCCA |
|  | Reverse primer | ACAATCGGAAAGAACACAGAACC |
| *R1B-14* | Forward primer | CCTGGTCATTTGGTTCATTTTCTGC |
|  | Reverse primer | CTGTGCTATCTTTGCTTACTTGCT |
| *NCL* | Forward primer | CAATCGGGGCACAGGAATC |
|  | Reverse primer | AGGAATGGAGGATAGAAATGAAGAT |
| *CPK18* | Forward primer | CCATAGAAGCCGATGACGAAC |
|  | Reverse primer | CCTTGCCTGGTTTAGAGAGAGT |
| *Actin* | Forward primer | CCCATCCCTACCATAACACCG |
|  | Reverse primer | TGACCTATTTTCACGCAGGCT |

**Table S2.** Sequencing quality and genome mapping for RNA-seq analysis.

| Sample | Total reads | Q20 | GC percent | Percentage of mapped reads |
| --- | --- | --- | --- | --- |
| Calcium glycinate-1 | 30789288 | 98.23% | 44.71% | 91.48% |
| Calcium glycinate-2 | 22306600 | 97.70% | 44.64% | 91.73% |
| Calcium glycinate-3 | 28973719 | 98.62% | 44.36% | 92.38% |
| Calcium glycinate/SPc-1 | 39303626 | 97.72% | 44.22% | 95.41% |
| Calcium glycinate/SPc-2 | 24210078 | 98.38% | 43.57% | 95.40% |
| Calcium glycinate/SPc-3 | 21842756 | 98.66% | 44.30% | 96.10% |

**Figure S1** Field layout to determine the bioactivity of SPc-delivered calcium glycinate.


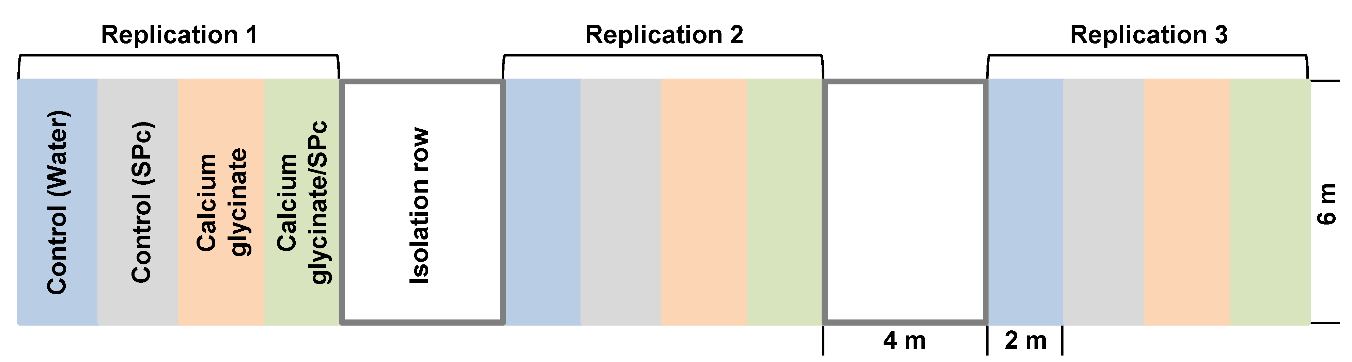


**Figure S2** Pearson correlation between collected samples.


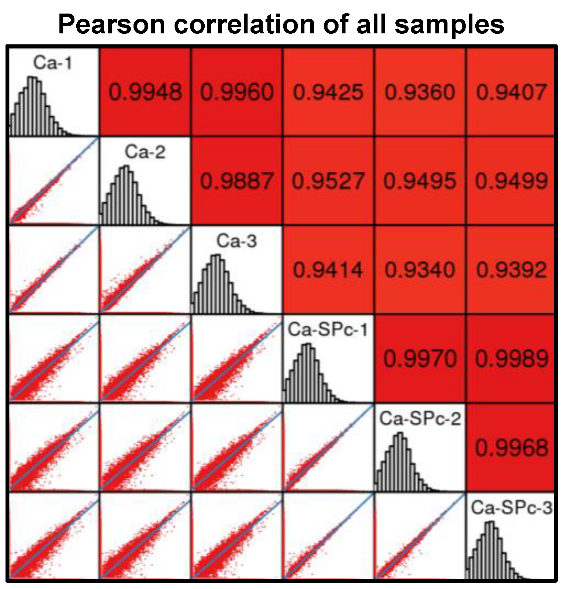


**Figure S3** Validation of differentially expressed genes using quantitative real-time PCR. The target gene expression was normalized to the abundance of *actin* gene. The asterisks indicate significant differences (Independent *t*-test. * *P* < 0.05, ****** *P* < 0.01 and ******* *P* < 0.001).


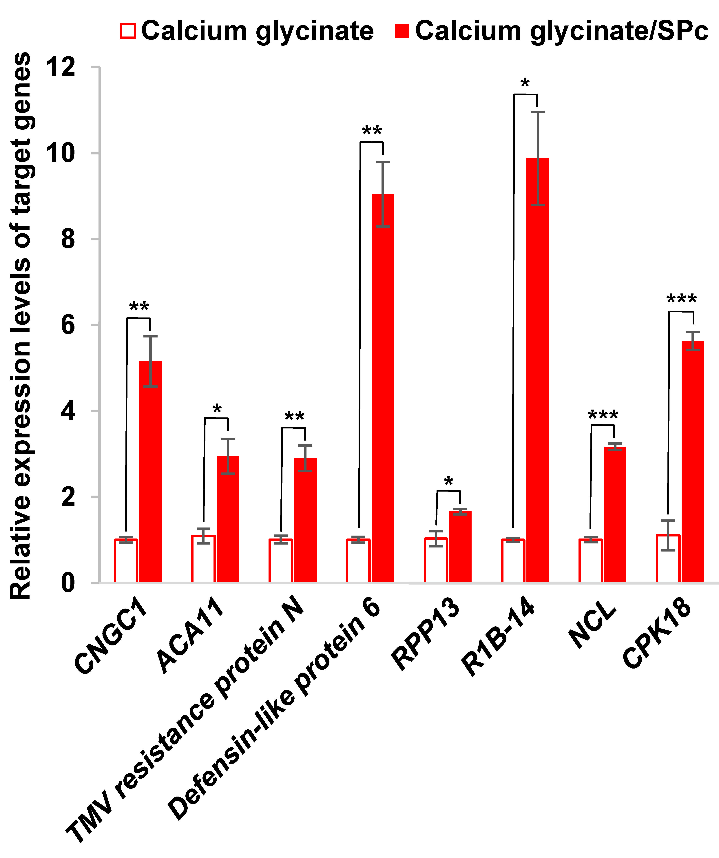

Supplement: Supplementary file 1 [file DataSheet_1.docx]
